# Poly[(μ_3_-biphenyl-3,3′-dicarboxyl­ato)(1,10-phenanthroline)cadmium]

**DOI:** 10.1107/S1600536811042085

**Published:** 2011-10-22

**Authors:** Yu-E Qiu

**Affiliations:** a Department of Chemistry, Dezhou University, Dezhou, Shandong 253023, People’s Republic of China

## Abstract

In the title compound, [Cd(C_14_H_8_O_4_)(C_12_H_8_N_2_)]_*n*_, the Cd^II^ ion is seven-coordinated in a distorted penta­gonal–bipyramidal coordination geometry by five O atoms from bridging biphenyl-3,3′-dicarboylate (dpda) ligands and two N atoms from a 1,10-phenanthroline (1,10-phen) ligand. In the crystal, dinuclear units with a Cd⋯Cd separation of 3.8208 (7) Å are observed. Each of these dinuclear units is bridged *via* 3,3′-bpda in a chelating/chelating and bridging fashion, generating a zigzag chain along the *c* axis. Neighboring chains are further packed *via* weak π–π inter­actions between inter­chain parallel 1,10-phen rings [centroid–centroid distance = 3.5197 (9) Å] into a three-dimensional supra­molecular architecture.

## Related literature

For the use of biphenyl­dicarboxyl­ato ligands in supra­molecular chemistry, see: Furukawa *et al.* (2008 ▶); Qu (2007 ▶); Zhu (2010 ▶).
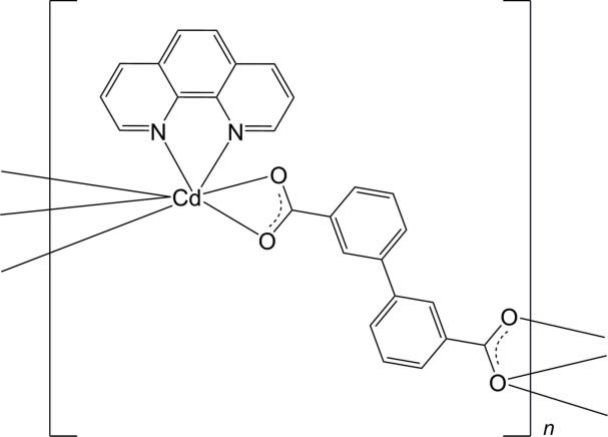


## Experimental

### 

#### Crystal data

[Cd(C_14_H_8_O_4_)(C_12_H_8_N_2_)]
*M* *_r_* = 532.81
Monoclinic, 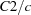

*a* = 26.1947 (17) Å
*b* = 9.7258 (5) Å
*c* = 21.2247 (14) Å
β = 127.411 (1)°
*V* = 4295.0 (5) Å^3^
*Z* = 8
Mo *K*α radiation
μ = 1.05 mm^−1^
*T* = 296 K
0.22 × 0.16 × 0.12 mm

#### Data collection

Bruker APEXII CCD area-detector diffractometer
Absorption correction: multi-scan (*SADABS*; Bruker, 2001 ▶) *T*_min_ = 0.804, *T*_max_ = 0.887
12984 measured reflections
4902 independent reflections
3799 reflections with *I* > 2σ(*I*)
*R*_int_ = 0.029

#### Refinement

*R*[*F* ^2^ > 2σ(*F* ^2^)] = 0.033
*wR*(*F* ^2^) = 0.075
*S* = 1.04
4902 reflections
298 parameters
20 restraints
H-atom parameters constrained
Δρ_max_ = 0.68 e Å^−3^
Δρ_min_ = −0.39 e Å^−3^

### 

Data collection: *APEX2* (Bruker, 2007 ▶); cell refinement: *SAINT* (Bruker, 2007 ▶); data reduction: *SAINT*; program(s) used to solve structure: *SIR97* (Altomare *et al.*, 1999 ▶); program(s) used to refine structure: *SHELXL97* (Sheldrick, 2008 ▶); molecular graphics: *SHELXTL* (Sheldrick, 2008 ▶); software used to prepare material for publication: *WinGX* (Farrugia, 1999 ▶).

## Supplementary Material

Crystal structure: contains datablock(s) global, I. DOI: 10.1107/S1600536811042085/im2322sup1.cif

Structure factors: contains datablock(s) I. DOI: 10.1107/S1600536811042085/im2322Isup2.hkl

Additional supplementary materials:  crystallographic information; 3D view; checkCIF report

## Figures and Tables

**Fig. 1.**
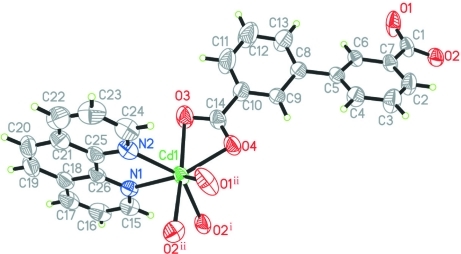
Anisotropic displacement ellipsoid plot of (I) at the 50% probability level. H atoms are represented by circles of arbitrary size. Symmetry code: (i)-x + 1, -y + 2, -z + 1; (ii)x, -y + 2, z - 1/2.

**Fig. 2.**
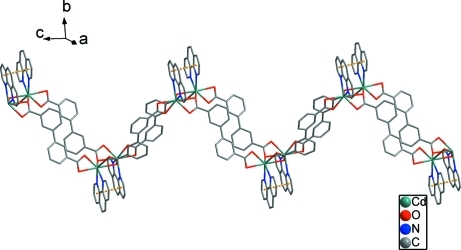
The one–dimensional zigzag chain structure of (I).

## supplementary crystallographic information

### Comment

Polycarboxylate ligands have been widely used to construct coordination polymers due to their versatile coordination modes. The use of biphenyldicarboxylic acid and its derivatives have been reported in literature (Qu, 2007; Furukawa *et al.*, 2008; Zhu, 2010). The title coordination polymer [Cd(C_14_H_8_O_4_)(C_12_H_8_N_2_)]_n_, (I), was obtained under hydrothermal conditions and herein its crystal structure is reported.

There is one Cd^II^ cation, one 3,3'-biphenyl-dicarboxylate anion (3,3'-bpda) and one 1,10-phenanthroline (1,10-phen) ligand observed in the asymmetric unit of (I). The Cd^II^ ion is seven coordinated in a distorted pentagonal bipyramidal coordination geometry by five O atoms (O1^ii^, O2^i^, O2^ii^, O3, O4) from bridging 3,3'-bpda with Cd—O bond lengths in the range of 2.258 (2)–2.515 (3) Å, two N atoms (N1, N2) from two 1,10-phen ligands with Cd—N bond lengths of 2.336 (3) and 2.368 (3) Å (Fig. 1). In the crystal structure of (I), dinuclear units with a Cd···Cd separation of 3.8208 (7) Å are observed. Each of these dinuclear units is bridged *via* 3,3'-bpda in a µ_1_η^1^:η^1^/µ_2_η^1^:η^2^ coordination mode into one dimensional zigzag chains. Parallel 1, 10-phen ligands are attached to the outside of the zigzag chain with centroid distances of 3.5197 (9) Å indicating weak π-π stacking interactions (Fig. 2). Neighboring chains are further packed *via* weak π-π interactions between interchain parallel 1,10-phen rings into the resulting three dimensional supramolecular architecture.

### Experimental

To a 16 ml Teflon-lined stainless steel vessel was loaded 3,3'-biphenyl-dicarboxylic acid (0.0242 g, 0.1 mmol), 1,10-phenanthroline (0.0198 g, 0.1 mmol), NaOH (0.0080 g, 0.2 mmol) and Cd(NO_3_)_2_ × 4H_2_O (0.0308 g, 0.1 mmol), then it was sealed and heated to 160 °C for 72 h. After being cooled down to room temperature at a rate of -5 °C/h, colorless block shaped crystals are obtained after filtration. Yield: 0.025 g (47% based on Cd).

### Refinement

All H atoms bonded to C atoms were added according to theoretical models, assigned isotropic displacement parameters and allowed to ride on their respective parent atoms [C—H = 0.93–0.97 Å and *U*_iso_(H) = 1.2*U*_eq_(C)].

### Crystal data

**Table:**

[Cd(C_14_H_8_O_4_)(C_12_H_8_N_2_)]	*F*(000) = 2128
*M**_r_* = 532.81	*D*_x_ = 1.648 Mg m^−^^3^
Monoclinic, *C*2/*c*	Mo *K*α radiation, λ = 0.71073 Å
Hall symbol: -C 2yc	Cell parameters from 4252 reflections
*a* = 26.1947 (17) Å	θ = 2.3–26.6°
*b* = 9.7258 (5) Å	µ = 1.05 mm^−^^1^
*c* = 21.2247 (14) Å	*T* = 296 K
β = 127.411 (1)°	Block, colorless
*V* = 4295.0 (5) Å^3^	0.22 × 0.16 × 0.12 mm
*Z* = 8	

### Data collection

**Table:**

Bruker APEXII CCD area-detector diffractometer	4902 independent reflections
Radiation source: fine-focus sealed tube	3799 reflections with *I* > 2σ(*I*)
graphite	*R*_int_ = 0.029
φ and ω scans	θ_max_ = 27.5°, θ_min_ = 2.0°
Absorption correction: multi-scan (*SADABS*; Bruker, 2001)	*h* = −33→34
*T*_min_ = 0.804, *T*_max_ = 0.887	*k* = −12→12
12984 measured reflections	*l* = −22→27

### Refinement

**Table:**

Refinement on *F*^2^	Primary atom site location: structure-invariant direct methods
Least-squares matrix: full	Secondary atom site location: difference Fourier map
*R*[*F*^2^ > 2σ(*F*^2^)] = 0.033	Hydrogen site location: inferred from neighbouring sites
*wR*(*F*^2^) = 0.075	H-atom parameters constrained
*S* = 1.04	*w* = 1/[σ^2^(*F*_o_^2^) + (0.0264*P*)^2^ + 5.2631*P*] where *P* = (*F*_o_^2^ + 2*F*_c_^2^)/3
4902 reflections	(Δ/σ)_max_ = 0.002
298 parameters	Δρ_max_ = 0.68 e Å^−^^3^
20 restraints	Δρ_min_ = −0.39 e Å^−^^3^

### Special details

**Table:**

Geometry. All e.s.d.'s (except the e.s.d. in the dihedral angle between two l.s. planes) are estimated using the full covariance matrix. The cell e.s.d.'s are taken into account individually in the estimation of e.s.d.'s in distances, angles and torsion angles; correlations between e.s.d.'s in cell parameters are only used when they are defined by crystal symmetry. An approximate (isotropic) treatment of cell e.s.d.'s is used for estimating e.s.d.'s involving l.s. planes.
Refinement. Refinement of *F*^2^ against ALL reflections. The weighted *R*-factor *wR* and goodness of fit *S* are based on *F*^2^, conventional *R*-factors *R* are based on *F*, with *F* set to zero for negative *F*^2^. The threshold expression of *F*^2^ > σ(*F*^2^) is used only for calculating *R*-factors(gt) *etc*. and is not relevant to the choice of reflections for refinement. *R*-factors based on *F*^2^ are statistically about twice as large as those based on *F*, and *R*- factors based on ALL data will be even larger.

### Fractional atomic coordinates and isotropic or equivalent isotropic displacement parameters (Å^2^)

**Table:**

	*x*	*y*	*z*	*U*_iso_*/*U*_eq_	
Cd1	0.576743 (12)	0.54986 (2)	0.356955 (13)	0.03981 (9)	
O4	0.58387 (13)	0.7247 (2)	0.43196 (14)	0.0562 (6)	
O2	0.53633 (12)	1.4416 (2)	0.71597 (14)	0.0510 (6)	
N1	0.55605 (13)	0.3153 (3)	0.35361 (14)	0.0391 (6)	
N2	0.66457 (14)	0.4227 (3)	0.38232 (16)	0.0462 (7)	
O1	0.60664 (16)	1.2882 (2)	0.80000 (16)	0.0743 (9)	
O3	0.63552 (15)	0.5452 (3)	0.50456 (15)	0.0715 (8)	
C8	0.63079 (16)	0.9542 (3)	0.62890 (18)	0.0419 (7)	
C22	0.7643 (2)	0.2526 (5)	0.4166 (2)	0.0690 (11)	
H22	0.7980	0.1969	0.4287	0.083*	
C15	0.50313 (17)	0.2623 (4)	0.33813 (19)	0.0493 (8)	
H15	0.4707	0.3218	0.3267	0.059*	
C9	0.61051 (15)	0.8739 (3)	0.56284 (18)	0.0384 (7)	
H9	0.5779	0.9068	0.5125	0.046*	
C6	0.60253 (15)	1.1531 (3)	0.67908 (18)	0.0378 (7)	
H6	0.6193	1.1044	0.7256	0.045*	
C25	0.66020 (16)	0.2840 (3)	0.38477 (18)	0.0429 (8)	
C7	0.57685 (16)	1.2827 (3)	0.66989 (18)	0.0398 (7)	
C5	0.60382 (15)	1.0940 (3)	0.62004 (18)	0.0372 (7)	
C21	0.70931 (19)	0.1938 (4)	0.4015 (2)	0.0540 (9)	
C26	0.60325 (16)	0.2285 (3)	0.37016 (17)	0.0410 (7)	
C24	0.71735 (19)	0.4739 (4)	0.3960 (2)	0.0607 (10)	
H24	0.7207	0.5686	0.3937	0.073*	
C14	0.61750 (17)	0.6662 (3)	0.4976 (2)	0.0462 (8)	
C10	0.63737 (18)	0.7474 (4)	0.5699 (2)	0.0510 (9)	
C23	0.7685 (2)	0.3906 (5)	0.4137 (2)	0.0713 (12)	
H23	0.8049	0.4303	0.4234	0.086*	
C2	0.5534 (2)	1.3575 (4)	0.6021 (2)	0.0577 (10)	
H2	0.5363	1.4447	0.5957	0.069*	
C1	0.57283 (18)	1.3394 (3)	0.7327 (2)	0.0475 (8)	
C3	0.5556 (2)	1.3028 (4)	0.5438 (2)	0.0668 (12)	
H3	0.5407	1.3539	0.4986	0.080*	
C17	0.5397 (2)	0.0336 (4)	0.3544 (2)	0.0605 (11)	
H17	0.5336	−0.0606	0.3544	0.073*	
C4	0.57985 (18)	1.1717 (4)	0.5526 (2)	0.0513 (9)	
H4	0.5801	1.1351	0.5123	0.062*	
C16	0.4936 (2)	0.1214 (4)	0.3381 (2)	0.0600 (10)	
H16	0.4555	0.0885	0.3270	0.072*	
C19	0.6478 (2)	−0.0034 (4)	0.3886 (2)	0.0646 (11)	
H19	0.6439	−0.0982	0.3901	0.078*	
C11	0.6841 (3)	0.6979 (5)	0.6435 (3)	0.0999 (17)	
H11	0.7014	0.6111	0.6491	0.120*	
C18	0.59693 (19)	0.0839 (3)	0.37142 (19)	0.0505 (9)	
C13	0.6786 (2)	0.9026 (5)	0.7021 (2)	0.0867 (16)	
H13	0.6926	0.9536	0.7470	0.104*	
C12	0.7065 (3)	0.7767 (6)	0.7108 (3)	0.118 (2)	
H12	0.7399	0.7446	0.7610	0.142*	
C20	0.7008 (2)	0.0496 (4)	0.4026 (2)	0.0659 (11)	
H20	0.7332	−0.0095	0.4135	0.079*	

### Atomic displacement parameters (Å^2^)

**Table:**

	*U*^11^	*U*^22^	*U*^33^	*U*^12^	*U*^13^	*U*^23^
Cd1	0.05906 (17)	0.02976 (12)	0.03860 (14)	−0.00476 (11)	0.03382 (12)	−0.00224 (10)
O4	0.0822 (18)	0.0413 (13)	0.0422 (14)	0.0027 (13)	0.0362 (14)	−0.0058 (11)
O2	0.0564 (15)	0.0473 (13)	0.0569 (15)	0.0043 (12)	0.0383 (13)	−0.0115 (11)
N1	0.0439 (15)	0.0375 (14)	0.0341 (14)	−0.0049 (12)	0.0227 (12)	0.0023 (11)
N2	0.0523 (17)	0.0492 (17)	0.0444 (16)	−0.0107 (14)	0.0332 (14)	−0.0059 (13)
O1	0.145 (3)	0.0429 (14)	0.0609 (17)	0.0272 (16)	0.0757 (19)	0.0103 (12)
O3	0.107 (2)	0.0565 (13)	0.0489 (9)	0.0251 (13)	0.0461 (13)	0.0007 (11)
C8	0.0485 (19)	0.0465 (18)	0.0372 (17)	0.0058 (16)	0.0295 (15)	−0.0008 (15)
C22	0.066 (3)	0.094 (3)	0.058 (2)	0.010 (3)	0.043 (2)	−0.002 (2)
C15	0.055 (2)	0.049 (2)	0.046 (2)	−0.0103 (17)	0.0320 (18)	0.0016 (16)
C9	0.0429 (18)	0.0410 (17)	0.0334 (16)	0.0023 (14)	0.0243 (14)	−0.0009 (13)
C6	0.0468 (18)	0.0352 (15)	0.0384 (17)	−0.0041 (14)	0.0295 (15)	−0.0034 (13)
C25	0.052 (2)	0.0461 (19)	0.0315 (17)	−0.0015 (16)	0.0260 (16)	−0.0051 (14)
C7	0.0506 (19)	0.0361 (16)	0.0413 (17)	−0.0006 (14)	0.0324 (16)	−0.0045 (14)
C5	0.0402 (17)	0.0390 (16)	0.0388 (17)	−0.0025 (13)	0.0273 (15)	−0.0064 (13)
C21	0.061 (2)	0.067 (2)	0.0387 (19)	0.0051 (19)	0.0325 (18)	−0.0048 (17)
C26	0.054 (2)	0.0372 (17)	0.0282 (16)	−0.0054 (15)	0.0231 (15)	−0.0034 (13)
C24	0.065 (3)	0.066 (3)	0.061 (2)	−0.016 (2)	0.044 (2)	−0.0065 (19)
C14	0.061 (2)	0.0433 (14)	0.0453 (19)	0.0021 (16)	0.0380 (18)	−0.0038 (15)
C10	0.068 (2)	0.0494 (19)	0.0386 (18)	0.0156 (18)	0.0343 (18)	0.0033 (15)
C23	0.056 (3)	0.103 (4)	0.070 (3)	−0.008 (2)	0.046 (2)	−0.003 (3)
C2	0.086 (3)	0.0426 (19)	0.056 (2)	0.0179 (19)	0.049 (2)	0.0063 (17)
C1	0.074 (2)	0.0332 (17)	0.053 (2)	−0.0027 (17)	0.048 (2)	−0.0059 (16)
C3	0.107 (3)	0.057 (2)	0.053 (2)	0.024 (2)	0.057 (2)	0.0156 (18)
C17	0.083 (3)	0.039 (2)	0.048 (2)	−0.019 (2)	0.034 (2)	−0.0024 (16)
C4	0.071 (2)	0.053 (2)	0.0431 (19)	0.0113 (18)	0.0417 (19)	−0.0005 (16)
C16	0.071 (3)	0.055 (2)	0.050 (2)	−0.024 (2)	0.035 (2)	−0.0018 (18)
C19	0.093 (3)	0.0386 (19)	0.055 (2)	0.006 (2)	0.041 (2)	−0.0048 (17)
C11	0.136 (4)	0.084 (3)	0.060 (3)	0.060 (3)	0.050 (3)	0.004 (2)
C18	0.072 (3)	0.0370 (17)	0.0343 (18)	−0.0015 (17)	0.0277 (18)	−0.0024 (14)
C13	0.112 (4)	0.089 (3)	0.037 (2)	0.051 (3)	0.034 (2)	−0.002 (2)
C12	0.150 (4)	0.109 (3)	0.056 (3)	0.073 (3)	0.042 (3)	0.006 (2)
C20	0.088 (3)	0.056 (2)	0.054 (2)	0.023 (2)	0.043 (2)	−0.0011 (19)

### Geometric parameters (Å, °)

**Table:**

Cd1—O4	2.258 (2)	C6—H6	0.9300
Cd1—N1	2.336 (3)	C25—C21	1.413 (5)
Cd1—N2	2.368 (3)	C25—C26	1.432 (4)
Cd1—O2^i^	2.368 (2)	C7—C2	1.380 (4)
Cd1—O1^ii^	2.388 (2)	C7—C1	1.505 (4)
Cd1—O2^ii^	2.497 (2)	C5—C4	1.387 (4)
Cd1—O3	2.515 (3)	C21—C20	1.423 (5)
Cd1—C14	2.731 (3)	C26—C18	1.419 (4)
O4—C14	1.245 (4)	C24—C23	1.406 (6)
O2—C1	1.271 (4)	C24—H24	0.9300
O2—Cd1^i^	2.368 (2)	C14—C10	1.509 (4)
O2—Cd1^iii^	2.497 (2)	C10—C11	1.360 (5)
N1—C15	1.320 (4)	C23—H23	0.9300
N1—C26	1.355 (4)	C2—C3	1.378 (5)
N2—C24	1.325 (4)	C2—H2	0.9300
N2—C25	1.358 (4)	C3—C4	1.386 (5)
O1—C1	1.238 (4)	C3—H3	0.9300
O1—Cd1^iii^	2.388 (2)	C17—C16	1.341 (6)
O3—C14	1.243 (4)	C17—C18	1.401 (6)
C8—C13	1.367 (5)	C17—H17	0.9300
C8—C9	1.397 (4)	C4—H4	0.9300
C8—C5	1.491 (4)	C16—H16	0.9300
C22—C23	1.352 (6)	C19—C20	1.336 (6)
C22—C21	1.393 (5)	C19—C18	1.428 (6)
C22—H22	0.9300	C19—H19	0.9300
C15—C16	1.393 (5)	C11—C12	1.398 (6)
C15—H15	0.9300	C11—H11	0.9300
C9—C10	1.380 (4)	C13—C12	1.380 (6)
C9—H9	0.9300	C13—H13	0.9300
C6—C7	1.385 (4)	C12—H12	0.9300
C6—C5	1.398 (4)	C20—H20	0.9300
			
O4—Cd1—N1	132.46 (9)	C4—C5—C6	117.3 (3)
O4—Cd1—N2	125.46 (10)	C4—C5—C8	120.7 (3)
N1—Cd1—N2	70.87 (9)	C6—C5—C8	122.0 (3)
O4—Cd1—O2^i^	88.24 (9)	C22—C21—C25	117.3 (4)
N1—Cd1—O2^i^	80.75 (9)	C22—C21—C20	123.4 (4)
N2—Cd1—O2^i^	145.46 (8)	C25—C21—C20	119.3 (4)
O4—Cd1—O1^ii^	87.04 (9)	N1—C26—C18	121.6 (3)
N1—Cd1—O1^ii^	140.34 (9)	N1—C26—C25	119.2 (3)
N2—Cd1—O1^ii^	83.53 (10)	C18—C26—C25	119.2 (3)
O2^i^—Cd1—O1^ii^	108.10 (10)	N2—C24—C23	122.6 (4)
O4—Cd1—O2^ii^	127.82 (8)	N2—C24—H24	118.7
N1—Cd1—O2^ii^	94.27 (8)	C23—C24—H24	118.7
N2—Cd1—O2^ii^	86.32 (8)	O3—C14—O4	121.7 (3)
O2^i^—Cd1—O2^ii^	76.36 (8)	O3—C14—C10	120.2 (3)
O1^ii^—Cd1—O2^ii^	53.28 (8)	O4—C14—C10	118.1 (3)
O4—Cd1—O3	53.83 (8)	O3—C14—Cd1	66.77 (18)
N1—Cd1—O3	88.58 (8)	O4—C14—Cd1	54.92 (16)
N2—Cd1—O3	86.12 (9)	C10—C14—Cd1	172.8 (2)
O2^i^—Cd1—O3	113.01 (9)	C11—C10—C9	118.9 (3)
O1^ii^—Cd1—O3	120.11 (10)	C11—C10—C14	119.8 (3)
O2^ii^—Cd1—O3	170.56 (9)	C9—C10—C14	121.2 (3)
O4—Cd1—C14	26.82 (9)	C22—C23—C24	119.6 (4)
N1—Cd1—C14	111.54 (9)	C22—C23—H23	120.2
N2—Cd1—C14	106.69 (10)	C24—C23—H23	120.2
O2^i^—Cd1—C14	101.72 (9)	C3—C2—C7	119.8 (3)
O1^ii^—Cd1—C14	104.46 (9)	C3—C2—H2	120.1
O2^ii^—Cd1—C14	153.63 (9)	C7—C2—H2	120.1
O3—Cd1—C14	27.01 (9)	O1—C1—O2	121.8 (3)
C14—O4—Cd1	98.26 (19)	O1—C1—C7	119.2 (3)
C1—O2—Cd1^i^	129.8 (2)	O2—C1—C7	119.0 (3)
C1—O2—Cd1^iii^	89.51 (19)	C2—C3—C4	120.0 (3)
Cd1^i^—O2—Cd1^iii^	103.50 (8)	C2—C3—H3	120.0
C15—N1—C26	118.5 (3)	C4—C3—H3	120.0
C15—N1—Cd1	125.3 (2)	C16—C17—C18	120.0 (3)
C26—N1—Cd1	116.2 (2)	C16—C17—H17	120.0
C24—N2—C25	117.8 (3)	C18—C17—H17	120.0
C24—N2—Cd1	126.5 (3)	C3—C4—C5	121.5 (3)
C25—N2—Cd1	115.8 (2)	C3—C4—H4	119.2
C1—O1—Cd1^iii^	95.4 (2)	C5—C4—H4	119.2
C14—O3—Cd1	86.2 (2)	C17—C16—C15	119.5 (4)
C13—C8—C9	117.5 (3)	C17—C16—H16	120.2
C13—C8—C5	121.0 (3)	C15—C16—H16	120.2
C9—C8—C5	121.4 (3)	C20—C19—C18	120.7 (4)
C23—C22—C21	119.9 (4)	C20—C19—H19	119.7
C23—C22—H22	120.1	C18—C19—H19	119.7
C21—C22—H22	120.1	C10—C11—C12	120.4 (4)
N1—C15—C16	123.1 (4)	C10—C11—H11	119.8
N1—C15—H15	118.5	C12—C11—H11	119.8
C16—C15—H15	118.5	C17—C18—C26	117.4 (4)
C10—C9—C8	122.1 (3)	C17—C18—C19	123.1 (4)
C10—C9—H9	118.9	C26—C18—C19	119.5 (4)
C8—C9—H9	118.9	C8—C13—C12	121.6 (4)
C7—C6—C5	121.5 (3)	C8—C13—H13	119.2
C7—C6—H6	119.3	C12—C13—H13	119.2
C5—C6—H6	119.3	C13—C12—C11	119.3 (4)
N2—C25—C21	122.8 (3)	C13—C12—H12	120.3
N2—C25—C26	117.9 (3)	C11—C12—H12	120.3
C21—C25—C26	119.4 (3)	C19—C20—C21	121.9 (4)
C2—C7—C6	119.8 (3)	C19—C20—H20	119.0
C2—C7—C1	120.3 (3)	C21—C20—H20	119.0
C6—C7—C1	119.9 (3)		
			
N1—Cd1—O4—C14	45.0 (3)	Cd1—N2—C24—C23	177.4 (3)
N2—Cd1—O4—C14	−51.1 (2)	Cd1—O3—C14—O4	0.5 (4)
O2^i^—Cd1—O4—C14	120.7 (2)	Cd1—O3—C14—C10	−178.2 (3)
O1^ii^—Cd1—O4—C14	−131.0 (2)	Cd1—O4—C14—O3	−0.5 (4)
O2^ii^—Cd1—O4—C14	−168.08 (19)	Cd1—O4—C14—C10	178.2 (3)
O3—Cd1—O4—C14	0.3 (2)	O4—Cd1—C14—O3	179.5 (4)
O4—Cd1—N1—C15	60.5 (3)	N1—Cd1—C14—O3	33.6 (3)
N2—Cd1—N1—C15	−178.5 (3)	N2—Cd1—C14—O3	−41.9 (2)
O2^i^—Cd1—N1—C15	−18.5 (2)	O2^i^—Cd1—C14—O3	118.2 (2)
O1^ii^—Cd1—N1—C15	−125.7 (3)	O1^ii^—Cd1—C14—O3	−129.4 (2)
O2^ii^—Cd1—N1—C15	−93.9 (2)	O2^ii^—Cd1—C14—O3	−158.9 (2)
O3—Cd1—N1—C15	95.1 (2)	N1—Cd1—C14—O4	−145.9 (2)
O4—Cd1—N1—C26	−118.5 (2)	N2—Cd1—C14—O4	138.5 (2)
N2—Cd1—N1—C26	2.46 (19)	O2^i^—Cd1—C14—O4	−61.3 (2)
O2^i^—Cd1—N1—C26	162.5 (2)	O1^ii^—Cd1—C14—O4	51.1 (2)
O1^ii^—Cd1—N1—C26	55.2 (3)	O2^ii^—Cd1—C14—O4	21.6 (3)
O2^ii^—Cd1—N1—C26	87.1 (2)	O3—Cd1—C14—O4	−179.5 (4)
O3—Cd1—N1—C26	−83.9 (2)	O4—Cd1—C14—C10	−12.9 (19)
O4—Cd1—N2—C24	−51.3 (3)	N1—Cd1—C14—C10	−158.8 (19)
N1—Cd1—N2—C24	179.7 (3)	N2—Cd1—C14—C10	126 (2)
O2^i^—Cd1—N2—C24	143.2 (3)	O2^i^—Cd1—C14—C10	−74 (2)
O1^ii^—Cd1—N2—C24	30.4 (3)	O1^ii^—Cd1—C14—C10	38 (2)
O2^ii^—Cd1—N2—C24	83.9 (3)	O2^ii^—Cd1—C14—C10	9(2)
O3—Cd1—N2—C24	−90.5 (3)	O3—Cd1—C14—C10	168 (2)
O4—Cd1—N2—C25	127.0 (2)	C8—C9—C10—C11	1.4 (6)
N1—Cd1—N2—C25	−2.0 (2)	C8—C9—C10—C14	−176.3 (3)
O2^i^—Cd1—N2—C25	−38.5 (3)	O3—C14—C10—C11	12.1 (6)
O1^ii^—Cd1—N2—C25	−151.3 (2)	O4—C14—C10—C11	−166.7 (4)
O2^ii^—Cd1—N2—C25	−97.8 (2)	O3—C14—C10—C9	−170.2 (4)
O3—Cd1—N2—C25	87.8 (2)	O4—C14—C10—C9	11.0 (5)
O4—Cd1—O3—C14	−0.3 (2)	C21—C22—C23—C24	0.5 (6)
N1—Cd1—O3—C14	−149.0 (2)	N2—C24—C23—C22	0.5 (6)
N2—Cd1—O3—C14	140.1 (2)	C6—C7—C2—C3	0.2 (6)
O2^i^—Cd1—O3—C14	−69.7 (2)	C1—C7—C2—C3	−178.0 (4)
O1^ii^—Cd1—O3—C14	59.9 (3)	Cd1^iii^—O1—C1—O2	−2.7 (4)
O2^ii^—Cd1—O3—C14	103.3 (5)	Cd1^iii^—O1—C1—C7	174.9 (3)
C26—N1—C15—C16	0.1 (5)	Cd1^i^—O2—C1—O1	−104.7 (4)
Cd1—N1—C15—C16	−178.9 (2)	Cd1^iii^—O2—C1—O1	2.6 (4)
C13—C8—C9—C10	−0.4 (5)	Cd1^i^—O2—C1—C7	77.7 (4)
C5—C8—C9—C10	176.5 (3)	Cd1^iii^—O2—C1—C7	−175.0 (3)
C24—N2—C25—C21	0.3 (5)	C2—C7—C1—O1	−163.1 (4)
Cd1—N2—C25—C21	−178.1 (2)	C6—C7—C1—O1	18.7 (5)
C24—N2—C25—C26	179.9 (3)	C2—C7—C1—O2	14.6 (5)
Cd1—N2—C25—C26	1.5 (3)	C6—C7—C1—O2	−163.6 (3)
C5—C6—C7—C2	−1.8 (5)	C7—C2—C3—C4	1.4 (6)
C5—C6—C7—C1	176.4 (3)	C2—C3—C4—C5	−1.5 (6)
C7—C6—C5—C4	1.6 (5)	C6—C5—C4—C3	0.0 (5)
C7—C6—C5—C8	−179.4 (3)	C8—C5—C4—C3	−178.9 (4)
C13—C8—C5—C4	149.9 (4)	C18—C17—C16—C15	0.2 (5)
C9—C8—C5—C4	−26.9 (5)	N1—C15—C16—C17	−0.1 (5)
C13—C8—C5—C6	−29.0 (5)	C9—C10—C11—C12	−2.7 (8)
C9—C8—C5—C6	154.2 (3)	C14—C10—C11—C12	175.1 (5)
C23—C22—C21—C25	−1.0 (5)	C16—C17—C18—C26	−0.3 (5)
C23—C22—C21—C20	179.4 (4)	C16—C17—C18—C19	−179.5 (3)
N2—C25—C21—C22	0.6 (5)	N1—C26—C18—C17	0.4 (5)
C26—C25—C21—C22	−179.0 (3)	C25—C26—C18—C17	−178.1 (3)
N2—C25—C21—C20	−179.8 (3)	N1—C26—C18—C19	179.6 (3)
C26—C25—C21—C20	0.6 (5)	C25—C26—C18—C19	1.1 (5)
C15—N1—C26—C18	−0.3 (4)	C20—C19—C18—C17	178.4 (4)
Cd1—N1—C26—C18	178.9 (2)	C20—C19—C18—C26	−0.7 (5)
C15—N1—C26—C25	178.2 (3)	C9—C8—C13—C12	0.7 (7)
Cd1—N1—C26—C25	−2.7 (3)	C5—C8—C13—C12	−176.2 (5)
N2—C25—C26—N1	0.8 (4)	C8—C13—C12—C11	−2.0 (10)
C21—C25—C26—N1	−179.6 (3)	C10—C11—C12—C13	3.0 (10)
N2—C25—C26—C18	179.3 (3)	C18—C19—C20—C21	0.3 (6)
C21—C25—C26—C18	−1.1 (4)	C22—C21—C20—C19	179.3 (4)
C25—N2—C24—C23	−0.9 (5)	C25—C21—C20—C19	−0.2 (6)

Symmetry codes: (i) −*x*+1, −*y*+2, −*z*+1; (ii) *x*, −*y*+2, *z*−1/2; (iii) *x*, −*y*+2, *z*+1/2.

## References

[bb1] Altomare, A., Burla, M. C., Camalli, M., Cascarano, G. L., Giacovazzo, C., Guagliardi, A., Moliterni, A. G. G., Polidori, G. & Spagna, R. (1999). *J. Appl. Cryst.* **32**, 115–119.

[bb2] Bruker (2001). *SADABS* Bruker AXS Inc., Madison, Wisconsin, USA.

[bb3] Bruker (2007). *APEX2* and *SAINT* Bruker AXS Inc., Madison, Wisconsin, USA.

[bb4] Farrugia, L. J. (1999). *J. Appl. Cryst.* **32**, 837–838.

[bb5] Furukawa, H., Kim, J., Ockwig, N. W., O’Keeffe, M. & Yaghi, O. M. (2008). *J. Am. Chem. Soc.* **130**, 11650–11661. 10.1021/ja803783c 18693690

[bb6] Qu, Z. (2007). *Chin. J. Inorg. Chem.* **23**, 1837–1839.

[bb7] Sheldrick, G. M. (2008). *Acta Cryst.* A**64**, 112–122. 10.1107/S0108767307043930 18156677

[bb8] Zhu, B.-Y. (2010). *Acta Cryst.* E**66**, m1214. 10.1107/S1600536810035269 PMC2983367 21587372

